# Statin therapy and neuropathy in type 1 diabetes: A cross‐sectional study

**DOI:** 10.1111/dom.16617

**Published:** 2025-07-24

**Authors:** Raabya Pasha, Anoushka Kamath, Zara Linn, Alise Kalteniece, Bilal Bashir, Jonathan D. Schofield, Rayaz Malik, Shazli Azmi, Maryam Ferdousi, Handrean Soran

**Affiliations:** ^1^ Division of Cardiovascular Sciences, Faculty of Biology, Medicine and Health The University of Manchester Manchester UK; ^2^ Manchester University NHS Foundation Trust Manchester UK; ^3^ NIHR/Wellcome Trust Clinical Research Facility Manchester UK; ^4^ Department of Diabetes, Endocrinology and Metabolism Manchester University NHS Foundation Trust Manchester UK; ^5^ Weill Cornell Medicine‐Qatar Doha Qatar

**Keywords:** diabetic neuropathy, lipid‐lowering therapy, pain, type 1 diabetes

## Abstract

**Aims:**

Dyslipidaemia contributes to the pathogenesis of diabetic peripheral neuropathy (DPN). While statins improve cardiovascular outcomes in diabetes, their potential neurotoxic effects remain debated. This study examined the impact of statin use on neuropathy in type 1 diabetes mellitus (T1DM).

**Materials and Methods:**

Participants with T1DM (*n* = 160) and healthy controls (*n* = 64) underwent symptom and clinical evaluation of DPN, cardiac autonomic neuropathy (CAN) and corneal confocal microscopy (CCM). T1DM participants were stratified by statin use (non‐statin: *n* = 68; statin treated: *n* = 92).

**Results:**

There were significant differences between the non‐statin and statin patients with T1DM and healthy controls for the diabetic neuropathy symptom score (DNS) (0.49 ± 0.14 vs. 0.90 ± 0.13 vs. 0.15 ± 0.13, *p* < 0.001), neuropathy disability score (NDS) (2.55 ± 0.29 vs. 3.67 ± 0.26 vs. 0.44 ± 0.28, *p* < 0.001), vibration perception threshold (13.68 ± 1.16 vs. 16.03 ± 1.03 vs. 6.05 ± 1.08, *p* < 0.001), corneal nerve fibre density (19.61 ± 1.04 vs. 19.02 ± 0.92 vs. 28.48 ± 0.97, *p* < 0.001), branch density (20.40 ± 2.21 vs. 21.39 ± 1.94 vs. 37.31 ± 2.05, *p* < 0.001), fibre length (11.97 ± 0.51 vs. 11.51 ± 0.45 vs. 16.55 ± 0.47, *p* < 0.001), DB‐HRV (26.27 ± 1.76 vs. 24.21 ± 1.51 vs. 30.18 ± 1.67, *p* = 0.033) and 30:15 ratio (1.32 ± 0.04 vs. 1.21 ± 0.03 vs. 1.15 ± 0.07, *p* = 0.033).

Despite the statin group being significantly older (*p* < 0.001) with a higher BMI (*p* = 0.001) and longer duration of diabetes (*p* < 0.001), statin‐treated patients showed no significant differences in most neuropathy measures, except DNS (*p* = 0.04), NDS (*p* = 0.009) and 30:15 ratio (*p* = 0.04).

**Conclusions:**

This study demonstrates that individuals with T1DM exhibit neuropathic symptoms and disability, increased vibration perception thresholds, corneal nerve fibre loss and evidence of CAN. However, statin therapy was associated with comparable measures of DPN and CAN, despite statin‐treated patients having a longer duration of diabetes and a higher BMI.

## 1 INTRODUCTION

Diabetic peripheral neuropathy (DPN) and cardiac autonomic neuropathy (CAN) affect at least 50% of patients with diabetes mellitus.^1^, ^2^ Older age, diabetes duration, and metabolic and vascular factors are associated with DPN.^1^, ^3^, ^4^ While the development and progression of DPN are reduced with improved glycaemic control,^5^ body mass index (BMI), total cholesterol and triglyceride levels, hypertension and smoking are key risk factors for DPN in type 1 diabetes mellitus (T1DM).^6^

Statins (3‐hydroxy‐3‐methylglutaryl‐CoenzymeA (HMG‐CoA) reductase inhibitors) are the most widely prescribed lipid‐lowering agents^7^ facilitating increased clearance of low‐density lipoprotein (LDL) particles^8^ and lowering of triglyceride levels.^9^ The National Institute for Health and Care Excellence (NICE) guidelines^10^ state that statins should be considered in patients with T1DM for primary prevention if they are aged 40 years or older, have had diabetes for longer than 10 years, have developed nephropathy or have other cardiovascular disease (CVD) risk factors.^10^

The impact of statin therapy on DPN remains controversial, especially as statin use has been associated with case reports of neuropathy, attributed to reduced production of farnesyl pyrophosphates, particularly ubiquinone,^11^ a key enzyme in the mitochondrial respiratory chain.^11^, ^12^ Statin use has been linked with the onset of peripheral neuropathy in some patients, although this development reversed upon discontinuation of the statin.^13^ It was also reported that statins, additional to their cardiovascular benefits, have been associated with improvements in neuropathy symptoms and nerve conduction^14^ as well as reducing the risk of developing peripheral neuropathy.^15^ However, sensory neuropathy development was seen in patients with hyperlipidaemia who were taking statins.^16^ On the other hand, Kristensen et al. reported that statin therapy does not significantly increase the risk of DPN,^17^ and Svendsen et al. found no clear association between statins and peripheral neuropathy,^18^ with other studies suggesting potential protective effects.^14^, ^19^, ^20^

While previous studies have produced conflicting results ranging from potential neurotoxicity to neuroprotection, only few have applied detailed and objective assessments of small and large fibre neuropathy in people with type 1 diabetes (T1DM). Given the widespread use of statins in this population, especially for primary prevention, clarifying their impact on neuropathy is essential to guide clinical decision making. In this study, we sought to address this important clinical question using detailed structural and functional assessments of small and large fibre neuropathy. We hypothesised that statin therapy does not worsen the severity of DPN or CAN in patients with T1DM.

## 2 METHODS

**FIGURE 1:**
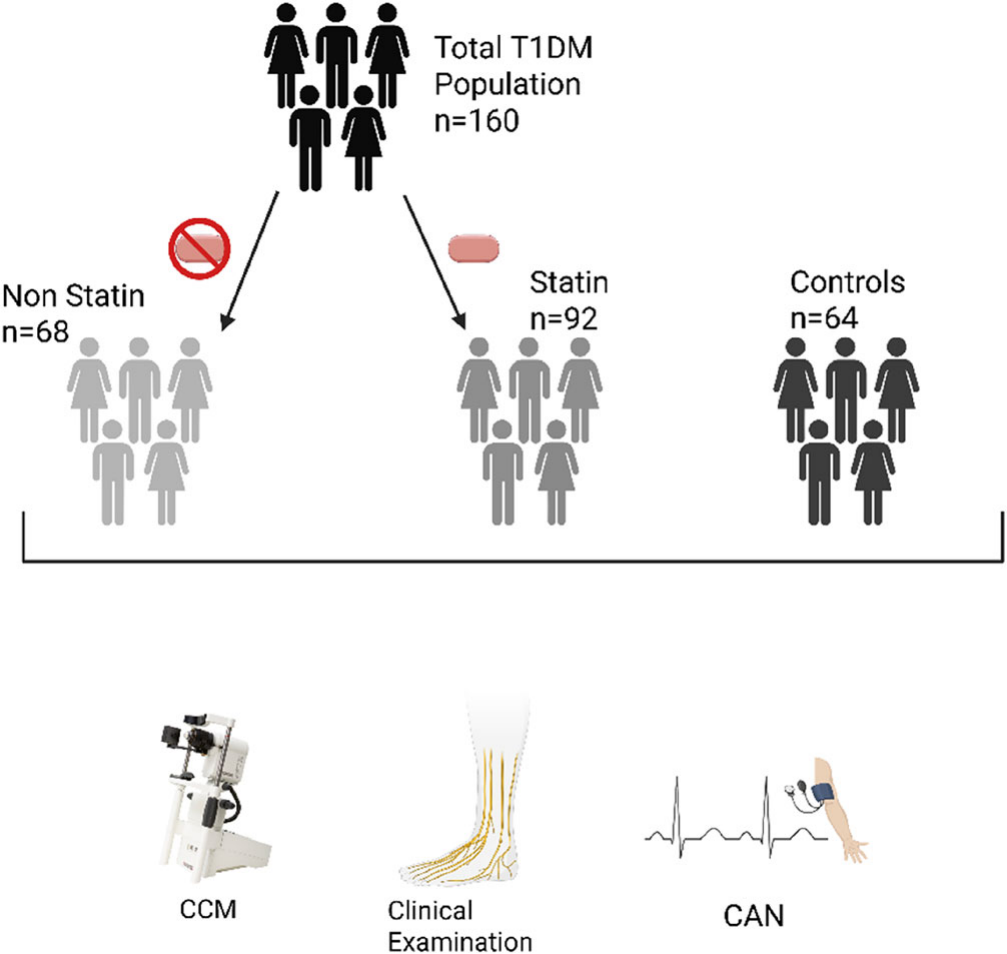
Diagram to show how patients were screened and placed into the different cohorts for the study. CCM, corneal confocal microscopy; CAN, cardiac autonomic neuropathy. Created in Biorender.com.

This cross‐sectional study recruited 160 patients with T1DM from outpatient clinics at the Manchester University NHS Foundation Trust and 64 healthy controls recruited from the University of Manchester and Manchester NHS Foundation Trust. Patients with T1DM were divided (Figure 1) into non‐statin users (*n* = 68) and statin users (*n* = 92). All patients who are currently taking statins or who have never used statins or other lipid‐lowering medications were included in the study.

All participants provided written informed consent before enrolment. The study was approved by the Greater Manchester Research Ethics Committee (REC No. 11/NW/0731, IRAS ID: 85208) and adhered to the Declaration of Helsinki for human research participants.

### 2.1 Demographic data

Demographic and clinical information was collected on the day of assessment, including age, sex, BMI, duration of diabetes, smoking status, alcohol intake and glycated haemoglobin (HbA1c). Participants were also asked whether they were currently taking or had previously taken statins or other lipid‐lowering therapies. This information was verified by reviewing each participant's electronic medical records to confirm current treatment and any history of lipid‐lowering medication use. Based on this information, patients with type 1 diabetes were categorised into two groups: those currently taking statins and those who had never received a statin or other lipid‐lowering treatment. Individuals who had previously taken these medications but were no longer doing so, were excluded. Data on diabetes‐related complications, including the presence of retinopathy, were also recorded. In addition, lipid profiles were obtained from recent blood test results and included total cholesterol, high‐density lipoprotein cholesterol (HDL‐C), low‐density lipoprotein cholesterol (LDL‐C) and triglyceride levels. These variables were used to characterise the study population and assess potential confounding factors.

### 2.2 Neuropathy Assessments

Neuropathy symptoms were evaluated with the neuropathy symptom profile (NSP) which assesses sensory, autonomic and motor symptoms with a score out of 38,^21^ diabetic neuropathy symptom score (DNS) which evaluates pain experienced over the last 2 weeks^22^ and the McGill pain questionnaire (descriptor, analogue score and index) which assesses the type and severity of neuropathic pain. The neuropathy disability score (NDS) assesses vibration perception, temperature sensation, pin prick perception and the Achilles reflex, with each test being scored as either present ‘0’ or absent/abnormal ‘1,’ except for Achilles reflex, where reinforcement was scored ‘1’ and absent/abnormal as ‘2’ to give a total score out of 10. Vibration perception threshold (VPT) was quantified using a neurothesiometer (Horwell, Scientific Laboratory Supplies, Wilford, Nottingham, UK) on both feet. Cardiac autonomic neuropathy (CAN) was assessed using the ZOE PPM3 autonomic nervous system monitoring device (Physio PS, Inc., New Hampshire, USA), which measures deep breathing heart rate variability (DB‐HRV), low‐frequency area:respiratory frequency area (LFA:RFA) ratio, expiration:inspiration (E:I) ratio, Valsalva ratio and 30:15 ratio. Corneal confocal microscopy (CCM) was performed using the Heidelberg Retinal Tomograph III Rostock Cornea Module (Heidelberg Engineering GmBH, Heidelberg, Germany). Images of the sub‐basal nerve plexus in the centre of the cornea, 6 images (3 per eye), were selected and analysed using ACCMetrics (University of Manchester) software following an established protocol.^23^

### 2.3 Statistical analysis

Statistical analysis was conducted using SPSS (Version 28.0.1.0, IBM Corporation, New York, USA). Shapiro–Wilk Normality test was used to determine the normality of the data. Data are presented as mean ± standard deviation (SD) for parametric variables. Data were adjusted for age using analysis of covariance (ANCOVA) with LSD correction, presented as mean ± standard error of the mean (SEM). HbA1c was not included as it did not differ significantly between the groups, and diabetes duration was considered to lie on the causal pathway.

Differences between groups were tested using paired‐sample t‐test for parametric data. Chi‐square test was used to investigate statistical differences in categorical data. Binary regression analysis was performed to assess the effect of statin use on DPN, based on the NDS, adjusting for age, BMI and duration of diabetes.

## 3 RESULTS

Table 1 summarises the clinical and demographic data in the T1DM non‐statin and statin group and healthy controls respectively. The statin group was significantly older (54.31 ± 13.07 vs. 32.65 ± 12.38 vs. 40.28 ± 15.07, *p* < 0.001), had a higher BMI (28.19 ± 5.16 vs. 26.13 ± 4.71 vs. 25.32 ± 4.80, *p* = 0.001) and longer duration of diabetes (34.96 ± 16.51 vs. 18.71 ± 12.42, *p* < 0.001) compared to the non‐statin group. The non‐statin group had a significantly higher HbA1c (66.78 ± 17.56 vs. 65.32 ± 13.60 vs. 36.56 ± 3.41, *p* < 0.001) than the statin user group and healthy controls. There was no significant difference in cholesterol levels, HbA1c, LDL levels, triglyceride levels, HDL levels, smoking and alcohol intake between the non‐statin and statin group.

**TABLE 1:** Demographic data for the control group and patients with T1DM: non‐statin and statin user groups, results displayed as mean ± SD.

	Controls Mean ± SD	Non‐statin Mean ± SD	Statin Mean ± SD	*p* value
Number of patients	64	68	92	
Age (years)	40.28 ± 15.07	32.65 ± 12.38^a^	54.31 ± 13.07^a^, ^b^	<0.001
Gender (sex): F, M (%)	60.9, 39.1	55.9, 44.1	44.6, 55.4	0.107
Duration of diabetes (years)	–	18.71 ± 12.42	34.96 ± 16.51	<0.001
Retinopathy (%)	0	33.8^a^	51.1^a^, ^b^	<0.001
Duration of statin use (months)	–	–	82.96 ± 66.14	
Statin type Atorvastatin (%) Pravastatin (%) Rosuvastatin (%) Simvastatin (%)	–	–	65.2 3.3 3.3 27.2	
BMI (kg/m^2^)	25.32 ± 4.80	26.13 ± 4.71	28.19 ± 5.16^a^, ^b^	0.001
HbA1c (mmol/mol)	36.56 ± 3.41	66.78 ± 17.56^a^	65.32 ± 13.60^a^	<0.001
Cholesterol (mmol/L)	4.86 ± 0.82	4.55 ± 0.87	4.32 ± 0.91^a^	0.002
HDL (mmol/L)	1.64 ± 0.37	2.08 ± 0.73^a^	1.96 ± 0.68^a^	<0.001
Triglyceride (mmol/L)	1.06 ± 0.37	1.18 ± 0.85	1.18 ± 0.78	0.584
LDL (mmol/L)	2.69 ± 0.75	1.99 ± 0.76^a^	1.85 ± 0.69^a^	<0.001
eGFR (mL/min/1.73 m^2^)	84.97 ± 8.64	86.67 ± 13.09	73.80 ± 23.43^a^, ^b^	<0.001
ACR (mg/mmol)	0.80 ± 1.72	17.85 ± 83.42	7.35 ± 14.30	0.164
Smoking (cigarettes/day)	0.32 ± 1.57	1.12 ± 3.56	1.35 ± 4.43	0.214
Alcohol (units/week)	4.28 ± 7.51	3.91 ± 5.01	5.77 ± 8.22	0.246

Abbreviations: ACR, urine albumin to creatinine ratio; BMI, body mass index; eGFR, estimated glomerular filtration rate; F, female; HbA1c, haemoglobin A1c; HDL, high‐density lipoprotein; LDL, low‐density lipoprotein; M, male.

^a^ Indicates significant difference (*p* < 0.05) compared to controls.

^b^ Indicates significant difference (*p* < 0.05) compared to non‐statin users.

Total cholesterol (4.86 ± 0.82 vs. 4.55 ± 0.87 vs. 4.32 ± 0.91, *p* = 0.002) and LDL (2.69 ± 0.75 vs. 1.99 ± 0.76 vs. 1.85 ± 0.69, *p* < 0.001) were higher, and HDL was significantly lower (1.64 ± 0.37 vs. 2.08 ± 0.73 vs. 1.96 ± 0.68, *p* < 0.001) in controls than in non‐statin and statin user groups. Retinopathy was significantly different across all groups (*p* < 0.001) and was prevalent mostly among the statin user group (51.1%) in comparison with the non‐statin group (33.8%) and the control (0%). Estimated glomerular filtration rate (eGFR) was significantly lower in the statin user group than in the non‐statin and control groups (73.80 ± 23.43 vs. 86.68 ± 13.09 vs. 84.97 ± 8.64, *p* < 0.001). However, the urine albumin to creatinine ratio (ACR) was not significantly different between the three groups.

### 3.1 Neuropathic symptoms

The neuropathy symptom profile (0.55 ± 0.73 vs. 2.74 ± 0.66 vs. 4.55 ± 0.61, *p* < 0.001), diabetic neuropathy symptom score (0.15 ± 0.13 vs. 0.49 ± 0.14 vs. 0.90 ± 0.13, *p* < 0.001) and McGill pain questionnaire (descriptor [0.17 ± 0.97 vs. 3.60 ± 1.05 vs. 4.16 ± 0.93, *p* = 0.006], analogue score [0.35 ± 0.46 vs. 2.83 ± 0.50 vs. 2.06 ± 0.44, *p* < 0.001] and index [0.12 ± 0.20 vs. 1.23 ± 0.22 vs. 1.02 ± 0.19, *p* < 0.001]) were significantly lower in the healthy control group than in the T1DM non‐statin and statin groups.

However, there were no significant differences in the neuropathy symptom profile (2.74 ± 0.66 vs. 4.55 ± 0.61, *p* = 0.067) and McGill pain questionnaire (descriptor [3.60 ± 1.05 vs. 4.16 ± 0.93, *p* = 0.713], analogue score [2.83 ± 0.50 vs. 2.06 ± 0.44, *p* = 0.284], index [1.23 ± 0.22 vs. 1.02 ± 0.19, *p* = 0.526]), and only the diabetic neuropathy symptom score was significantly lower (0.49 ± 0.14 vs. 0.90 ± 0.13, *p* = 0.040) in the T1DM non‐statin group than in the statin group.

### 3.2 Peripheral Neuropathy

**FIGURE 2:**
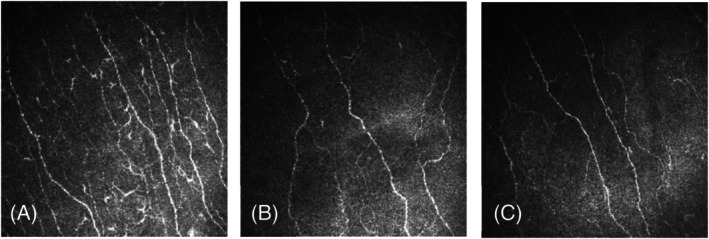
CCM images of the sub‐basal nerve plexus in a healthy control (A) and patients with T1DM: non‐statin user (B) and statin user (C).

**FIGURE 3:**
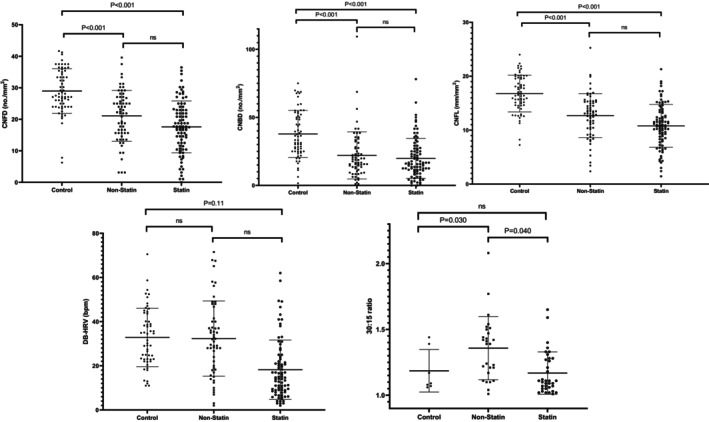
Graphs (top row) showing the CCM parameters (CNFD, CNBD, CNFL) in the healthy controls and patients with T1DM: non‐statin and statin groups. Graphs (bottom row) displaying the significantly different cardiac autonomic neuropathy results (DB‐HRV and 30:15 ratio) in the healthy controls and T1DM patients: non‐statin and statin user groups. CNFD, corneal nerve fibre density; CNBD, corneal nerve branch density; CNFL, corneal nerve fibre length; DB‐HRV, deep breathing heart rate variability.

The neuropathy disability score (0.44 ± 0.28 vs. 2.55 ± 0.29 vs. 3.67 ± 0.26, *p* < 0.001) and vibration perception threshold (6.05 ± 1.08 vs. 13.68 ± 1.16 vs. 16.03 ± 1.03, *p* < 0.001) were significantly lower in the control group compared to the T1DM non‐statin and statin user group. CCM parameters (Figure 2) (corneal nerve fibre density [CNFD] [28.48 ± 0.97 vs. 19.61 ± 1.04 vs. 19.02 ± 0.92, *p* < 0.001], corneal nerve branch density [CNBD] [37.31 ± 2.05 vs. 20.40 ± 2.21 vs. 21.39 ± 1.94, *p* < 0.001] and corneal nerve fibre length [CNFL] [16.55 ± 0.47 vs. 11.97 ± 0.51 vs. 11.51 ± 0.45, *p* < 0.001]) were more dense and had a longer length in the healthy control group compared with the T1DM non‐statin and statin user group (Table 2, Figure 3).

**TABLE 2:** Neuropathy assessments in controls compared to T1DM non‐statin and statin user groups.

	Control Mean ± SEM	Non‐statin Mean ± SEM	Statin Mean ± SEM	*p* value
NSP (/38)	0.55 ± 0.73	2.74 ± 0.66^a^	4.55 ± 0.61^a^	<0.001
DNS (/4)	0.15 ± 0.13	0.49 ± 0.14	0.90 ± 0.13^a^, ^b^	<0.001
NDS (/10)	0.44 ± 0.28	2.55 ± 0.29^a^	3.67 ± 0.26^a^, ^b^	<0.001
VPT (Hz)	6.05 ± 1.08	13.68 ± 1.16^a^	16.03 ± 1.03^a^	<0.001
10 g monofilament (/10)	9.83 ± 0.24	8.62 ± 0.26^a^	8.34 ± 0.25^a^	<0.001
McGill Pain Descriptor (/45)	0.17 ± 0.97	3.60 ± 1.05^a^	4.16 ± 0.93^a^	0.006
McGill Pain Analogue (/10)	0.35 ± 0.46	2.83 ± 0.50^a^	2.06 ± 0.44^a^	<0.001
McGill Pain Index (/5)	0.12 ± 0.20	1.23 ± 0.22^a^	1.02 ± 0.19^a^	<0.001
DB‐HRV (bpm)	30.18 ± 1.67	26.27 ± 1.76	24.21 ± 1.51^a^	0.033
LFA:RFA	1.74 ± 0.64	2.30 ± 0.31	2.63 ± 0.29	0.448
E:I ratio	1.09 ± 0.06	1.23 ± 0.03^a^	1.16 ± 0.03	0.086
Valsalva ratio	1.10 ± 0.15	1.38 ± 0.07	1.36 ± 0.07	0.228
30:15 ratio	1.15 ± 0.07	1.32 ± 0.04^a^	1.21 ± 0.03^b^	0.033
CNFD (no./mm^2^)	28.48 ± 0.97	19.61 ± 1.04^a^	19.02 ± 0.92^a^	<0.001
CNBD (no./mm^2^)	37.31 ± 2.05	20.40 ± 2.21^a^	21.39 ± 1.94^a^	<0.001
CNFL (mm/mm^2^)	16.55 ± 0.47	11.97 ± 0.51^a^	11.51 ± 0.45^a^	<0.001

*Note*: Results were adjusted for age using ANCOVA (LSD correction).

Abbreviations: CNBD, corneal nerve branch density; CNFD, corneal nerve fibre density; CNFL, corneal nerve fibre length; DB‐HRV, deep breathing heart rate variability; DNS, diabetic neuropathy symptom; E, expiration; I, inspiration; LFA, low‐frequency area; RFA, respiratory; NDS, neuropathy disability score; NSP, neuropathic symptom profile; VPT, vibration perception threshold.

^a^ Indicates significant difference compared to controls (*p* < 0.05).

^b^ Indicates significant difference compared to non‐statin users (P < 0.05).

However, vibration perception threshold (13.68 ± 1.16 vs. 16.03 ± 1.03, *p* = 0.164), corneal nerve fibre density (19.61 ± 1.04 vs. 19.02 ± 0.92, *p* = 0.694), corneal nerve branch density (20.40 ± 2.21 vs. 21.39 ± 1.94, *p* = 0.754) and corneal nerve fibre length (11.97 ± 0.51 vs. 11.51 ± 0.45, *p* = 0.534) were comparable, and only NDS (2.55 ± 0.29 vs. 3.67 ± 0.26, *p* = 0.009) was significantly lower in the non‐statin group than in statin group.

### 3.3 Cardiac Autonomic Neuropathy

Deep breathing heart rate variability (DB‐HRV) (30.18 ± 1.67 vs. 26.27 ± 1.76 vs. 24.21 ± 1.51, *p* = 0.033) was higher in the healthy controls than in T1DM non‐statin and statin groups, with no difference between the T1DM non‐statin and statin user groups (Figure 3). The 30:15 ratio (1.15 ± 0.07 vs. 1.32 ± 0.04 vs. 1.21 ± 0.03, *p* = 0.033) was significantly lower in the healthy control than in the T1DM non‐statin and statin user groups, and the 30:15 ratio (1.32 ± 0.04 vs. 1.21 ± 0.03, *p* = 0.04) was significantly higher in the non‐statin group than in the statin group (Figure 3). LFA:RFA (1.74 ± 0.64 vs. 2.30 ± 0.31 vs. 2.63 ± 0.29, *p* = 0.448), E:I ratio (1.09 ± 0.06 vs. 1.23 ± 0.03 vs. 1.16 ± 0.03, *p* = 0.086) and Valsalva ratio (1.10 ± 0.15 vs. 1.38 ± 0.07 vs. 1.36 ± 0.07, *p* = 0.228) did not differ significantly between the control and T1DM non‐statin and statin user groups.

### 3.4 Regression analysis

Binary regression analysis was carried out to assess the impact of different factors including statin use on the development of DPN by dividing the patients with T1DM into those with (NDS ≥3) and without (NDS <3) neuropathy.^24^ Age (*r* = 1.09) was a significant predictor for DPN (*p* < 0.001), indicating that for each additional year in age, the odds of developing DPN increased by 8.8%. BMI (*r* = 1.14) was also a significant predictor for the development of DPN (*p* = 0.004), indicating that for each unit increase in BMI, there was a 14.1% increase in the odds of DPN. Although statin use (*r* = 0.71) was associated with a 28.6% reduced risk of developing DPN, this result was not statistically significant (*p* = 0.52), suggesting that further studies with larger sample sizes are needed. To confirm these findings, a sensitivity analysis was conducted with additional covariates, including sex, cholesterol and triglycerides. The results reinforced the primary conclusion: statin use remained non‐significant in predicting neuropathy (OR = 0.90, 95% CI [0.24, 3.31], *p* = 0.868). In this more comprehensive model, age and BMI persisted as the only significant predictors (*p* = 0.002 and *p* = 0.023 respectively), supporting the robustness of the initial conclusion that, in this cohort, statin therapy was not significantly associated with the odds of having DPN.

### 3.5 Statistical power

A post hoc power analysis was conducted using the corneal nerve fibre density (CNFD) data to assess whether the study was adequately powered to detect differences between the groups. Based on the observed means (statin group: 19.02 fibres/mm^2^; non‐statin group: 19.61 fibres/mm^2^), standard deviations (0.92 and 1.04 respectively) and sample sizes (*n* = 92 and *n* = 68), the calculated power was 0.96 at a significance level of 0.05. This indicates that the study was sufficiently powered to detect a clinically meaningful difference in CNFD between the groups.

## 4 DISCUSSION

There are currently no disease‐modifying therapies for DPN. This study shows that statin use may afford protection from DPN and CAN in patients with T1DM. Hyperlipidaemia is associated with the development of DPN in both T1DM^6^ and T2DM.^4^, ^25^ However, some studies have reported that statins may increase the risk of neuropathy, others report no association, and some report a protective effect.^25^, ^26^, ^27^ Indeed, epidemiological studies have reported a higher incidence of idiopathic peripheral neuropathy in patients treated with lipid‐lowering drugs^28^ with a 1.3‐fold increased risk in patients taking a statin.^29^ Gaist et al. reported a 4‐ to 14‐fold increased risk of developing idiopathic polyneuropathy in non‐diabetic patients taking statins.^30^ In a case series of seven patients prescribed a statin, patients developed neuropathic symptoms in their hands and feet and had no reflexes.^31^ Additionally statins may cause idiosyncratic somatic and autonomic neuropathy in patients with diabetes.^32^ Jeyam et al. previously reported an association between long‐term statin use and symptoms of DPN in patients with T1DM.^33^ However, Svendsen et al. reported no significant impact on nerve conduction or intraepidermal nerve fibre density among statin users,^18^, ^34^ and other studies report no association between statins and peripheral neuropathy^35^ or idiopathic small fibre neuropathy.^34^ A systematic review and meta‐analysis concluded that there was no increase in the risk of developing DPN in patients taking statins.^36^ A large cohort study of 59 255 new statin users, 75 528 prevalent users and 124 842 non‐users followed over a median of 6.2 years reported a comparable incidence of DPN across all three groups.^17^ The American Heart Association concluded that there was no evidence for a causal relationship between statin treatment and peripheral neuropathy.^35^ A further meta‐analysis found no increase in risk of peripheral neuropathy in patients treated with statins.^37^

This is the first large study to conduct a detailed assessment of both neuropathic symptoms and objective functional and structural measures of large and small fibre neuropathy. We show that older, heavier patients with a longer duration of T1DM and therefore with major risk factors for DPN treated with statins have a comparable degree of neuropathy to patients with T1DM who are younger, weigh less and have a shorter duration of diabetes. Statin use has been associated with a reduced odds for developing idiopathic sensory neuropathy.^15^ Indeed, Hernández‐Ojeda et al. showed an improvement in neuropathic symptoms and peroneal nerve conduction velocity in patients with DPN after treatment with Rosuvastatin.^14^ Additionally, a population‐based study from Denmark reported a lower cumulative incidence of DPN or gangrene of the foot in statin users compared to non‐users.^20^ Furthermore, regression analysis in our study has revealed that age and BMI were significantly associated with a higher odds ratio of patients developing DPN, while statin use was associated with a reduced risk of DPN.

A key limitation of this study is its cross‐sectional design, which restricts our ability to draw definitive conclusions about the long‐term effects of statin therapy on the development or progression of neuropathy. Although the study was sufficiently powered for primary outcomes, such as corneal nerve fibre density (CNFD), it may have been underpowered to detect smaller or subgroup‐specific differences across other measures. In addition, although we documented the type and duration of statin use, the sample sizes for individual statins were too small to allow for meaningful subgroup analyses. As such, we were unable to explore potential differences in neuropathy outcomes between specific statins. Given the lack of consistent evidence in the literature regarding statin‐specific effects on nerve function, we treated statin use as a single exposure variable. Nevertheless, future studies with larger and more balanced groups may be better positioned to investigate whether different statins exert distinct effects on peripheral and autonomic neuropathy.

## 5 CONCLUSION

Our findings suggest that statin use in older patients with T1DM does not worsen neuropathy severity, despite their longer duration of diabetes and higher BMI, both recognised risk factors for neuropathy. While no significant protective effect was observed, the absence of neuropathy progression in a group with greater baseline risk at least supports the safety of statins in patients with type 1 diabetes and at high risk of developing DPN.

These findings are reassuring for clinicians managing patients with T1DM who require statins for cardiovascular risk reduction and suggest a possible disease modifying effect of statins on DPN in T1DM. However, further research is warranted to explore the benefits of statin therapy in a longitudinal study, and indeed, a randomised trial is warranted using objective end points to provide valuable insights into the role of statins in the management of DPN in T1DM.

## CONFLICT OF INTEREST STATEMENT

HS: Received personal fees from Amgen, Akcea, Synageva, NAPP, Novartis, Takeda, Sanofi, Pfizer and Kowa and research grants and donations from Akcea, Pfizer, MSD, Amgen, Genzyme‐Sanofi, Synageva, Amryt, Synageva and Alexion. JS: Consultancy and/or speaker honoraria from Amarin, Astra Zeneca, Daiichi‐Sankyo, Eli Lilly, Novartis, Roche, Sanofi; unrestricted educational grants from Astra Zeneca, Daiichi‐Sankyo, Eli Lilly, Novo Nordisk. RAM: Received personal fees from Novo Nordisk, Eli Lilly, Sanofi Aventis, Proctor and Gamble and Pfizer. SA: Speaker honoraria from Astra Zeneca, Eli Lilly, Menarini, Novo Nordisk, Sanofi. RP, AKam, ZL, AKal, BB, and MF has no competing interest.

## Data Availability

The data that support the findings of this study are available from the corresponding author, upon reasonable request. The data are not publicly available due to ethical and privacy restrictions relating to participant confidentiality.
